# *IFT81* as a Candidate Gene for Nonsyndromic Retinal Degeneration

**DOI:** 10.1167/iovs.16-19133

**Published:** 2017-05

**Authors:** Rachayata Dharmat, Wei Liu, Zhongqi Ge, Zixi Sun, Lizhu Yang, Yumei Li, Keqing Wang, Kandace Thomas, Ruifang Sui, Rui Chen

**Affiliations:** 1 Department of Molecular and Human Genetics, Baylor College of Medicine, Houston, Texas, United States; 2 Human Genome Sequencing Center, Baylor College of Medicine, Houston, Texas, United States; 3 Department of Ophthalmology, Peking Union Medical College Hospital, Peking Union Medical College, Chinese Academy of Medical Sciences, Beijing, China; 4 Department of Structural and Computational Biology & Molecular Biophysics, Baylor College of Medicine, Houston, Texas, United States; 5 Department of Biochemistry and Molecular Biology, Baylor College of Medicine, Houston, Texas, United States; 6 Program of Developmental Biology, Baylor College of Medicine, Houston, Texas, United States

**Keywords:** IFT-B complex, *IFT81*, cilia, cone rod dystrophy

## Abstract

**Purpose:**

IFT81, a core component of the IFT-B complex, involved in the bidirectional transport of ciliary proteins, has been recently implicated in syndromic ciliopathies. However, none of the IFT-B core complex proteins have been associated with nonsyndromic retinal dystrophies. Given the importance of ciliary transport in photoreceptor function and structural maintenance, we sought to investigate the impact of IFT (intraflagellar transport) mutations in nonsyndromic retinopathies.

**Methods:**

Whole exome sequencing was performed on 50 cone-rod dystrophy (CRD) patients that were previously screened for mutations in known retinal disease genes. The impact of candidate mutation was studied using in vitro cell system and in vivo zebrafish assay to determine the pathogenicity of the variant.

**Results:**

Compound heterozygous mutations in *IFT81*, including one nonsense (c.1213C>T, p.R405*) and one missense variant (c.1841T>C, p.L614P), were identified in a nonsyndromic CRD proband. Extensive functional analyses of the missense variant in cell culture and zebrafish strongly suggests its pathogenic nature. Loss of IFT81 impairs ciliogenesis and, interestingly, the missense variant displayed significantly reduced rescue of ciliogenesis in the *IFT81* knockdown in vitro system. Consistently, dramatic reduction of rescue efficiency of the *ift81* mutant zebrafish embryo by mRNA with the missense variant was observed, further supporting its pathogenicity.

**Conclusions:**

Consistent with the function of the IFT-B complex in the maintenance of photoreceptor cilium, we report a case of mutations in a core IFT-B protein, IFT81. This represents the first report of mutations in *IFT81* as a candidate gene for nonsyndromic retinal dystrophy, hence expanding the phenotype spectrum of IFT-B components.

Primary cilia are slender hair-like protuberances present on the cell surface, functioning as sensory antennae to relay extracellular signals to the cell. Mutations in genes encoding for ciliary proteins can cause structural or functional defects in the cilia and have been implicated in a number of developmental and degenerative diseases collectively called ciliopathies. Phenotypes observed in these diseases can range from syndromic multisystem pathologies affecting the central nervous system, heart, kidney, skeletal system, and retina, to nonsyndromic ciliopathies affecting a single organ system.^1,2^ This variation in clinical features is a phenotypic reflection of the function of underlying gene and the severity of mutant alleles carried by the patient. For example, although complete loss-of-function mutations in *IQCB1* and *CEP290* cause systemic Senior-Loken syndrome (MIM #266900) and Bardet-Biedl syndrome (MIM #209900), respectively, hypomorphic mutations in these two genes leads to Leber's congenital amaurosis, an early onset nonsyndromic retinopathy (MIM #204000).^3–6^

Approximately one-third of nonsyndromic retinal dystrophies are due to mutations in ciliary-related genes.^2^ In such cases, a modified sensory cilium named the connecting cilium, which connects and mediates a bi-directional protein transport between the inner and outer segments of the photoreceptors, is affected.^7^ This protein transportation process is carried out by a highly conserved molecular machinery called the intraflagellar transport (IFT) complex, consisting of both IFT-A and IFT-B complex. Anterograde transport along the cilium is carried out by the IFT-B complex, which consists of at least 16 subunits, 9 of which form a salt-stable core complex (IFT81, -88, -52, -46, -27, -74, -70, -25, and -22).^8–12^ Genetic studies indicate that the core of the IFT-B complex is functionally conserved in all ciliated organisms from *Chlamydomonas reinhardtii* to vertebrates.^13^ Consistent with these studies, two core subunits of the IFT-B complex (IFT88, and -27) have been previously associated with syndromic ciliopathies,^14–17^ indicating the importance of these proteins in maintaining ciliary function in humans. Furthermore, mutations in peripheral members of the IFT-B complex, such as IFT172, have been previously linked to not only syndromic ciliopathies but also nonsyndromic retinal dystrophies.^18^ Interestingly, however, none of the core complex components have been associated with nonsyndromic retinal pathology to date in humans.

As a member of the IFT-B core complex, IFT81 forms the backbone of the core complex along with IFT72/74. The N-terminal calponin homology domain of IFT81 interacts with the N-terminal domain of IFT72 to form a tubulin binding module required for transportation of tubulin during ciliogenesis.^8,19,20^ Like other IFT-B core members, mutations in *IFT81* have been observed to cause syndromic ciliopathies featuring renal medullary cysts, paraxial polydactyly, early onset rod-cone dystrophy, cerebellar atrophy, and intellectual disability.^21^ However, thus far, the association between mutations in *IFT81* and nonsyndromic human disease has not been reported.

In our study, we investigated the genetics of cone-rod dystrophy (CRD), a progressive inherited retinal disorder by collecting and performing whole-exome sequencing of a cohort of CRD patients whose mutations remain undetermined after known retinal disease-related gene panel screening.^22^ Among them, one proband carrying bi-allelic mutations in *IFT81* was identified. Both in vitro and in vivo functional assays of the putative mutant allele indicate that it negatively impacts protein function and is likely to be pathogenic. Therefore, our finding provides the first report linking the core IFT-B protein, IFT81, to nonsyndromic retinopathy.

## Materials and Methods

### Subject and Clinical Evaluation

The proband was diagnosed with CRD and recruited at the Department of Ophthalmology, Peking Union Medical College Hospital (PUMCH). Ophthalmic examinations were performed including best-corrected visual acuity (BCVA) testing, fundus examination, optical coherence tomography (OCT, 3D OCT-2000 Spectral Domain; Topcon, Tokyo, Japan), autofluorescence (AF, Spectralis HRA+OCT; Heidelberg, Germany) and electroretinogram (ERG, RetiPort ERG system; Roland Consult, Wiesbaden, Germany). Informed consent was obtained from the patient for this study. Blood samples were obtained from the patient and her parents. This study adhered to the Declaration of Helsinki and was approved by the Institutional Review Board PUMCH.

### Whole-Exome Sequencing and Bioinformatics Analysis

Genomic DNA sample (approximately 1 μg) was sheared into 300- to 500-bp-long fragments and repaired with a single adenine base added to the 3′ ends using Klenow exonuclease. Illumina bar-coded adapters were ligated to the ends, and DNA fragments were PCR amplified. The DNA was then captured using the NimblegenSeqCap EZ Human Exome Library v.2.0 following the manufacturer's protocols for whole-exome sequencing. Captured libraries were sequenced on the Illumina HiSeq 2000 (Illumina, San Diego, CA, USA) to generate 100-bp paired-end reads according to the manufacturer's protocol.

Reads were mapped to the human reference genome hg19 using the Burrows-Wheeler Aligner.^23^ Base quality recalibration, local realignment, and variant calling were performed as previously described.^24^ Because CRD is a rare Mendelian disease, variants with a normal control population allele frequency higher than 0.5% (for a recessive model) or 0.1% (for a dominant model) in public or internal control databases were excluded.^24^ Databases used for this purpose include the 1000 Genome Database, dbSNP135 (National Center for Biotechnology Information, http://www.ncbi.nlm.nih.gov/SNP, in the public domain), the National Heart, Lung, and Blood Institute (NHLBI) Exome Sequencing database (http://evs.gs.washington.edu/EVS, in the public domain), the National Institute of Environmental Health Sciences (NIEHS) Exome Sequencing database (http://evs.gs.washington.edu/niehsExome, in the public domain), and an internal control database of 997 exomes. We also retrieved variant frequency from the Exome Aggregation Consortium (ExAC) database.^25^ Pathogenicity of missense variants was predicted using SIFT,^26^ Polyphen2,^27^ LRT,^28^ MutationTaster,^29^ and MutationAssessor^30^ as previously described.^24^

### Sanger Sequencing and Segregation Test

For each putative mutation, a 500-bp flanking sequence at both sides was obtained from the University of California, Santa Cruz (UCSC) genome browser (hg19 assembly). Primer 3^31^ was used to design a pair of primers for generating a 400- to 600-bp PCR product containing the variant for Sanger validation. After PCR amplification, the amplicons were sequenced on an ABI 3730xl. Family members of the patient were also Sanger-sequenced to test allele segregation with the disease.

### DNA Constructs

Human *IFT81* cDNA in pENTR-221 vector was obtained from Ken Scott and subcloned into the p3XFLAG-myc-CMV-14 vector obtained from G. Pazour. The patient-specific missense variant at exon 18 (c.1841T>C, p.L614P) was created using the Agilent's QuikChange XL-II site-directed mutagenesis kit. GIPZ human *IFT81* shRNA (GE Dharmacon) was obtained through the Baylor College of Medicine CBASS-shRNA library core facility (shRNA1: CATCTATCATTTCCCGTAA, shRNA2: CCTTTAGGAAGAACTATAA). The shRNAmir is coexpressed with Turbo GFP as a bi-cistronic transcript allowing the visual marking of shRNA-mir–expressing cells. To avoid shRNA silencing of the exogenous mRNA, two silent mutations per seed sequence were introduced in *IFT81* wild-type and c.1841T>C mutant vectors (Supplementary Fig. S3).

### In Vivo Zebrafish Functional Experiments

Capped mRNA from the human cDNA construct of the wild-type and c.1841T>C mutant *IFT81* was synthesized using Invitrogen's mMESSAGE mMACHINE Kit (T7). RNA was purified using Zymo's RNA Clean and Concentrator column. Zebrafish rescue experiments were performed on embryos of progeny from *ift81^hi409tg/+^* crosses (parental zebrafish were heterozygous mutants because homozygous mutant is lethal). Embryos were injected at the one-cell stage with 1 nL (50 ng/μL) of human wild-type and c.1841T>C mutant IFT81 mRNA tagged with C-myc and flag. Approximately 100 embryos were injected for each group (with four biological repeats). The phenotype was scored at days post fertilization 3.5. The *P* value was calculated from 2-way ANOVA analysis. Western blotting was performed on day 0 (hours post fertilization 6.0) and day 1 (hours post fertilization 26.0) whole embryo lysate. Anti–C-myc antibody (MMS-150p; Covance, Biologend, San Diego, CA, USA) was used to detect C-myc-IFT81 protein with bactin as a loading control (ab8227; Abcam, Cambridge, MA, USA). Experiments were designed and conducted in adherence to the ARVO Statement for the Use of Animals in Ophthalmic and Vision Research.

### Cell Culture, Transfection, and Ciliary Induction

hTERT-RPE1 and HEK 293T cells were cultured in 1:1 DMEM: F12 or DMEM, respectively, supplemented with 10% FBS. For transfection purpose, hTERT-RPE1 cells on coverslips were transfected with DNA constructs using Viafect chemical transfection reagent (Promega, Madison, WI, USA). For rescue experiments, short hairpin RNA (shRNA) and shRNA-resistant *IFT81* wild-type/mutant cDNA constructs were cotransfected at a 1:1 ratio. Following plasmid transfection, the formation of primary cilia was induced 14 hours after transfection by serum starvation (DMEM-F12 supplemented with 0.3% FBS) for 48 hours. For protein analysis, HEK 293T cells were transfected using MIRUS *transit*-293T reagents using the manufacturer's protocol.

### Immunofluorescence and Imaging

For immunofluorescence staining, hTERT RPE-1 cells were fixed, permeabilized, and blocked as previously described.^32^ Primary antibodies against *α*-acetylated tubulin (clone 6-11B-1, 1:500; Sigma-Aldrich Corp., St. Louis, MO, USA), green fluorescent protein (GFP; 1:500; Invitrogen, Carlsbad, CA, USA), and Flag (1:300, F7425; Sigma-Aldrich Corp.,) were used along with AlexaFluor 488 anti-mouse, Cy3 anti-rabbit, and Cy5 anti-mouse secondary antibodies, respectively, (Jackson Immunolabs, Bar Harbor, ME, USA). Cells were counterstained with 4′,6-diamidino-2-phenylindole (1 μg/mL). Representative images were acquired on an Axio observer Z.1 inverted fluorescence microscope with apotome.2 based optical sectioning and structured illumination, on a 60× EC plan-neofluar objective (Carl Zeiss International, Dublin, CA, USA). For cell counting, image stacks were taken with a z-distance of 0.5 μm and projected as a maximal intensity image using Zeiss Zen 2 core software (Carl Zeiss International). Three biological replicates were scored with 100 cells per replicate for all test panels using images taken on a 40× EC plan-neofluar objective (Carl Zeiss International).

See Supplementary Methods for protein analysis and statistical analysis.

## Results

### Identification of *IFT81* Mutations as a Candidate for CRD

**Figure 1:**
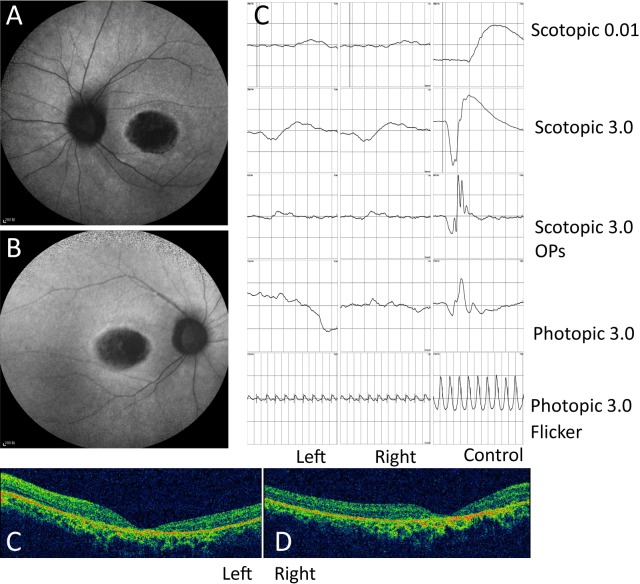
Fundus autofluorescence, ERG, and OCT tests display degeneration of macular region and loss of visual response in the proband. (**A**, **B**) Left and right fundus autofluorescence images display oval shaped macular hypofluorescence with a hyperfluorescent ring characteristic of CRD. (**C**) Scotopic and photopic ERGs: both rod and cone responses are significantly reduced, with more severe cone function loss. (**D**, **E**) Left and right eye OCT images display thinning of whole retina layers in the macular area observed in both eyes.

We performed whole-exome sequencing on a group of 50 unsolved CRD patients to discover candidate novel genes underlying the disease. One proband, a 22-year-old female, who displayed a progressive decrease in vision with photophobia since age 12, had impaired color vision and poor visual acuity (left eye, 0.05; right eye, 0.06). The proband lacked any skeleto-developmental defects previously reported including postaxial polydactyly. Ultrasound (B-scan) of the various organs including liver, kidney, and pancreas presented normal structure of the liver and kidneys. Blood routine, liver function, renal function, and blood fat tests were all in normal functional range (Supplementary Dataset S2: Test report), suggesting a lack of any syndromic defects. Fundus images display oval shaped macular hypofluorescence with a hyperfluorescent ring, which are characteristic pigmented lesions found in CRD. In addition, ERG results displayed reduced responses in both rod and cone cells, with more severe cone function loss (Fig. 1A–C). Finally, we observed thinning of retina layers in the macular area by OCT (Figs. 1D, 1E). Other than retina defects, the patient did not display an additional clinically syndromic phenotype.

**Figure 2:**
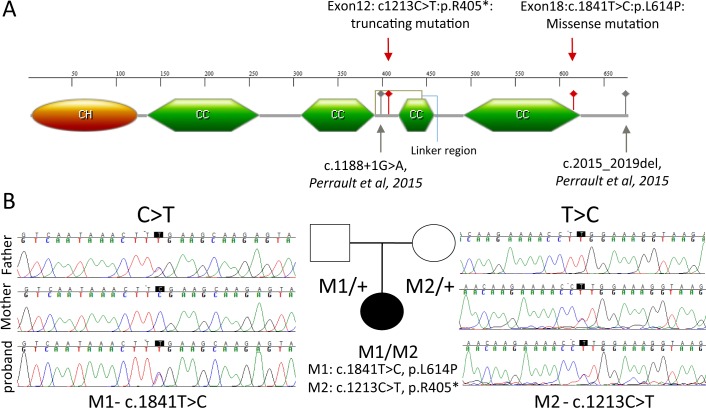
Candidate pathogenic compound heterozygous mutations in *IFT81* of a CRD proband: (**A**) IFT81 protein displaying putative domains and localization of proband's mutation within these domains. The nonsense mutation lies in the linker region,^8^ whereas the missense mutation lies in the last CC domain (CH, calponin homology domain; CC, coiled coil domain). (**B**) Sanger sequencing shows segregation of the missense (c.1841T>C, p.L614P) mutation and the nonsense (c.1213C>T, p.R405*) mutation within the family.

Because no mutations were identified during the initial screen of all known retinal disease genes, the proband was subjected to whole-exome sequencing. With mean sequence coverage of 90×, a total of 105,490 variants were initially identified. On filtering as described in the Materials and Methods section, 329 rare variants in 305 genes that affect protein coding remained. Among them, only four genes containing bi-allelic rare variants in their coding region were predicted to be damaging, including *FAM186A, PKHD1L1, IRS1,* and *IFT81* (Supplementary Table S1). *FAM186A* and *PKHD1L1* genes were excluded from the candidate list as homozygous loss of function mutations have been observed in a large number of individuals in the ExAC control cohort.^25^ Furthermore, *IRS1* was excluded from further analysis as *IRS1* has been previously linked to type 2 diabetes without retinal degeneration phenotype.^33^ As a result, *IFT81* is the best candidate gene with one nonsense (c.1213C>T, p.R405*) and one missense variant (c.1841T>C, p.L614P) identified in the proband (NM_001143779). Sanger sequencing and segregation analysis of the proband's family members showed that these two alleles were inherited separately from each parent and hence present *in trans* in the proband (Fig. 2A). Both variants are extremely rare as they have not been found in the public or in our internal control databases.

Based on current annotation, four different transcription isoforms encoding for three different types of proteins have been observed in the human *IFT81* gene. In the human retina, the long isoform of 676 amino acid encoded by the third transcript isoform (NM_001143779) is expressed at a much higher level (fpkm-6.99156) than that of the other two isoforms of 431 and 366 amino acids, respectively (NM_031473: fpkm-0.30852; ENSG00000122970: undetectable). The mutant alleles were found in the 12th and the 18th exons of *IFT81*, affecting the protein sequence of the long isoform. Because the variant in the 12th exon (c.1213C>T, p.R405*) is a nonsense variant, it results in early truncation of the protein. Furthermore, the transcript containing the variant is likely subjected to nonsense-mediated decay. The second allele is a missense variant in the 18th exon (c.1841T>C, p.L614P). This missense variant is rare in the human population and likely to negatively impact the protein function as it is predicted to be deleterious by all five computational prediction algorithms (Supplementary Table S1).

### Mutant (c.1841T>C) *IFT81* Fails to Rescue Ciliogenesis Defect In Vitro

**Figure 3:**
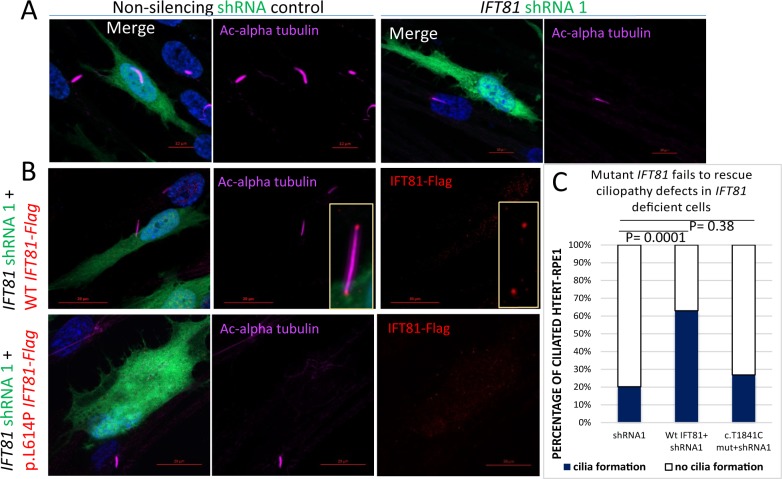
c.1841T>C-*IFT81* mutant expression leads to a ciliogenesis defect in hTERT-RPE overexpression systems. (**A**) Transient knockdown of endogenous *IFT81* in hTERT-RPE1 cells leads to loss of ciliogenesis marked by acetylated α-tubulin. Representative images showing presence and absence of ciliogenesis (marked by acetylated α-tubulin) in cells expressing nonsilencing shRNA (*left*) and *IFT81* targeting shRNA1 (*right*). (**B**) Representative images displaying significant (*P* = 0.0001) rescue of ciliogenesis in cells expressing shRNA1 by coexpression of WT *IFT81* (*top*). Coexpression of mutant *IFT81* does not significantly (*P* = 0.38) rescue ciliogenesis (*bottom*). (**C**) Quantification of cells coexpressing shRNA1 and WT or mutant *IFT81* and corresponding ciliated cell percentage.

To assess the effect of the missense variance identified in the patient on IFT81 protein function, the hTERT-RPE1 cell was used as the model system. The hTERT-RPE1 is an immortalized cell line derived from human retinal pigment epithelial cells capable of forming cilia. Consistent with the previous report that IFT81 is required for cilia assembly in the cell, knockdown of endogenous *IFT81* by shRNA (sequence: Materials and Methods and Supplementary Materials) leads to ciliogenesis defects. Specifically, on serum starvation, only 21% of shRNA-transfected cells grow cilia compared with 60% in wild-type cells (Fig. 3). To test whether the knockdown phenotype can be rescued by overexpression of *IFT81* cDNA, shRNA-resistant (Supplementary Fig. S3) wild-type and mutant *IFT81* cDNAs carrying the c.1841T>C mutation were cloned into mammalian expression vectors with Flag-tag at its N terminus and cotransfected with shRNA. In cells expressing both exogenous WT *IFT81* and shRNA, *IFT81*::FLAG was localized at the base and tip of the cilia as expected (Fig. 3A). Furthermore, 60% of cells displayed cilia as observed in wild-type cells, indicating the wild-type *IFT81* cDNA construct is capable of complete rescue of ciliogenesis. In contrast, in cells cotransfected with mutant *IFT81* cDNA along with shRNA, only 28% cells contain cilia, which is not significantly different from shRNA knockdown cells (*P* = 0.38). This indicates that the mutant cDNA fails to rescue the ciliogenesis defect in cells with *IFT81* knockdown (Fig. 3B). To confirm whether this difference in rescue efficiency between wild-type and mutant cDNA is not caused by a difference in protein level, Western blot was carried out. As shown in Supplementary Figure S2C, similar levels of protein are detected for the wild-type and mutant alleles, indicating the mutation does not affect protein translation and stability. In addition, a similar phenotype was observed when two different shRNA targeting endogenous *IFT81* (shRNA1, Fig. 3B; shRNA2, Supplementary Figs. S3A, S3B) were used. In both cases, the mutant flag tagged L614P-IFT81 protein displays localization signal throughout the cellular cytoplasm. Taken together, the (c.1841T>C) *IFT81* allele does not rescue the ciliogenesis defect due to endogenous *IFT81* knockdown, suggesting it is indeed deleterious to the protein function.

### *IFT81* c.1841T>C Mutation Fails to Rescue Ciliary Defects In Vivo

**Figure 4:**
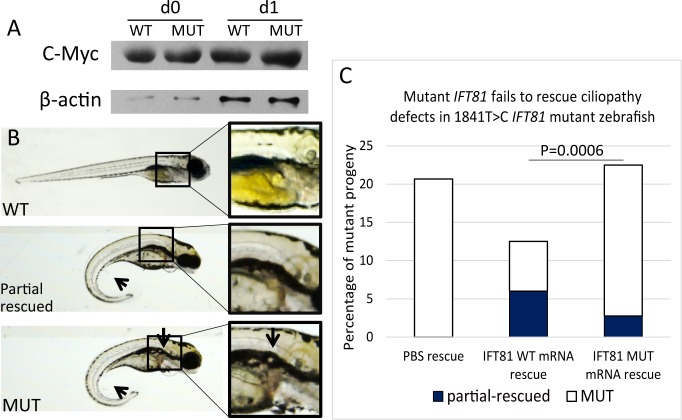
*IFT81* mutant in *ift81*^hi409tg/hi409tg^ background displays ciliary defects: functional assay of the human *IFT81* mutation in zebrafish *ift81*^hi409tg/+^ in-crosses. (**A**) Zebrafish embryos were injected with 50 pg human *IFT81*-C-Myc-flag or human c.1841T>C-*IFT81* mutant-C-Myc-flag mRNA, Western blotting was performed using an anti–C-Myc antibody, β-actin was used as a control (day 0:6 hours after injection; day 1: 28 hours after injection). (**B**) Bright field images of day 3.5 wild-type like embryo (WT), partial rescued embryo (without kidney cyst but with post-vent increased curvature), and mutant embryo (with postvent increased curvature and kidney cyst; see the *arrows* and *detailed inset* in the *box*). (**C**) Phenotype distribution of zebrafish embryos injected with 50 pg *IFT81* mRNA (WT), *IFT81* mutant mRNA (MUT), and noninjected control displaying a significant (***P = 0.0006) reduction in the rescue potential of mutant *IFT81* in comparison to WT.

To further assess in vivo whether the missense (c.1841T>C) mutation is detrimental to IFT81 function, we performed a rescue experiment using *ift81* mutant zebrafish. It has been reported that *ift81* mutant zebrafish embryos exhibit ciliary defects including spine curvature and kidney cyst formation.^34^ To first assess whether this p.L164P missense variant affects *IFT81* protein expression, we performed a Western blot on zebrafish embryos injected with wild-type or mutant *IFT81* mRNA. Consistent with the result obtained from the cell line as described above, mutant *IFT81* is expressed at similar levels to wild type, indicating that the variant does not affect protein level (Fig. 4A). To determine whether c.1841T>C*-IFT81* could rescue the ciliopathy defects, we injected wild-type or mutant mRNA into embryos resulting from *ift81^hi409tg/+^* crosses. As expected, these crosses produced about 25% of the *ift81^hi409tg/hi409tg^* mutant embryos, which were further assessed for rescue potential of the mutant allele. A significant rescue effect (*P* = 0.0006) was observed when wild-type *IFT81* mRNA was injected, with only 7% fish displaying ciliary defects. In contrast, injection of c.1841T>*C-IFT81* mRNA was unable to rescue the phenotype, as 21% of injected embryos display ciliary defects, similar to the proportion of noninjected control embryos (∼25%) (Fig. 4C). In addition, partial rescue in a small proportion of fish injected with wild-type *IFT81* mRNA has also been observed (7%). These fish display normal kidney morphology but still exhibit increased spinal curvature in the postvent (Figs. 4B, 4C). Therefore, *IFT81* mRNA with a c.1841T>C point mutation has a significantly lower rescue potential than that of the wild-type mRNA (*P* = 0.0006), consistent with the idea that the allele identified in the proband is likely to be pathogenic (Fig. 4C).

In summary, the p. L614P missense negatively impacts the function of IFT81 in both in vitro and in vivo assays. Together with the observation that the loss-of-function mutation in *IFT81* results in a severe syndromic defect in the patient while our proband only exhibits retina defects, the missense variant identified in this study is likely to be a hypomorphic allele, thereby exhibiting pathogenic effects.

## Discussion

In this report, we identified mutations in *IFT81* as candidates for a recessive form of nonsyndromic retinopathy. This is the first report highlighting the importance of a core IFT-B complex protein in retinal ciliopathy. The proband harbors a truncating mutation in exon 12 and a missense mutation in exon 18 acting *in trans*. Because the allele containing the nonsense truncating (c.1213C>T, p.R405*) mutation is likely subjected to nonsense mediated decay (NMD), the only remaining functional allele contains a nonsynonymous missense mutation leading to the substitution of leucine with a proline residue in the protein. The in silico predictions suggest that this substitution is functionally deleterious, supported by both in vivo and in vitro functional studies. Specifically, the c.1841T>C mutant allele displayed a significantly lower potential for rescuing the ciliary defects in *IFT81*-knockout hTERT-RPE cells and zebrafish, in comparison to wild type, supporting the functionally deleterious nature of this allele. Together, this provides compelling evidence supporting the pathogenicity of the variant according to American College of Medical Genetics and Genomics (ACMG) guidelines^35^ and classifies the variant as likely pathogenic allele class II. Due to limitations of exome sequencing, however, it is possible that larger deletions and structural aberrations in known retinitis pigmentosa genes may be missed. With exception of this caveat, given the functional evidence presented along with a lack of other potentially explanatory mutations, the c.1841T>C mutation in *IFT81* is the likely candidate for inherited retinal dystrophy in this proband.

Ciliopathies are known to have characteristically variable phenotypes caused by different mutations in the same gene, depending on the exact nature of the patients' alleles. For example, it has been reported that mutations in IFT-A core components, specifically, IFT140, IFT144, and IFT-B peripheral component IFT172^18,36,37^, can cause both syndromic and nonsyndromic cases of retinal dystrophies. Similarly, mutations in *IFT81* have also previously been associated with a syndromic disease featuring severe skeletal anomalies (polydactyly), nephronophthisis leading to renal abnormalities, intellectual disability, and retinal degeneration.^21^ Retinal pathology was observed in only one of the two patients displaying abnormal ocular movements, hemeralopia, and poor vision with altered scotopic and photopic ERG. The variation between the syndromic phenotype reported by Perrault et al.^21^ and the isolated retinal phenotype observed by our group is likely due to the nature of the identified mutations and their different corresponding impact on IFT81 function (Fig. 2). Due to the importance of IFT81 in maintaining the stability of the IFT-B complex, and by extension ciliary structure and functions, severe mutations leading to complete loss of IFT81 function may not be tolerated in nature and hence potentially lead to severe developmental defects. Indeed, it has been shown that loss of the C-terminal domain renders the IFT81 protein unstable, resulting in no observable assembly of IFT-B components in vitro.^38^ Severe, but not complete, loss-of-function mutations, like the in-frame exon loss and extension of IFT81 C-terminal end by 11 amino acids reported by Perrault et al. can lead to a syndromic phenotype.^21^ Therefore, it is plausible that a milder mutation, such as the missense mutation reported in this study, is likely to be a hypomorphic allele, thereby leading to nonsyndromic ciliary defects.

A simple model explaining the nonsyndromic clinical manifestation is that the retina is more sensitive to the IFT81 activity. The likely cause is that IFT complexes in the retina are involved in massive protein transportation from the protein synthesizing inner segment to the photoreceptive outer segment of the photoreceptor cells. This protein transport process is highly active and is crucial for proper photoreceptor architecture, outer-segment turnover, as well as phototransduction, hence rendering photoreceptors more sensitive to a decrease in function of the IFT complex.

It is possible that IFT81 function specifically in the retina is affected by the c.1841T>C mutation, for example, by altering/weakening its interactions with other retinal specific proteins. A similar case has been previously observed for ciliary proteins such as C21orf2, where hypomorphic mutations specifically impact its interaction with SPATA7, a retinal ciliopathy protein^39^. However, given the observation that the mRNA with c.1841T>C mutation cannot rescue the ciliary defect in hTERT-RPE cells or in a zebrafish model, it is likely that the defect is caused by the mutant allele. The c.1841T>C mutation allele might have a negative impact on IFT81 function, among other possibilities, due to problems in protein folding, defective localization, or reduced IFT81 interaction with other IFT-B proteins. In our efforts to further understand the mechanism, we investigated whether the c.1841T>C missense allele can weaken the interaction between IFT81 with other IFT-B components. However, the c.1841T>C mutant allele does not significantly alter the interaction of IFT81 with IFT52/46 subcomplex (Supplementary Fig. S5). This suggests that rather than interfering directly with protein interaction, this allele possibly affects protein localization. However, further biochemical characterization is necessary to investigate interactions with other IFT-B subunits and its effect on the kinetics of IFT-B transport.

In conclusion, our findings highlight *IFT81* as a candidate for inherited retinal dystrophy, thereby implying the importance of a core IFT-B protein, IFT81, in the human retina. This report represents the first link between *IFT81* and nonsyndromic retinal dystrophy, expanding the phenotypic spectrum of IFT-B core members as a potential cause of nonsyndromic retinal dystrophy in humans.

## Supplementary Material

Supplement 1

Supplement 2
