# Supplementary material for: IFT81 as a Candidate Gene for Nonsyndromic Retinal Degeneration
Source: Invest Ophthalmol Vis Sci. 2017 May;58(5):2483–90. doi: 10.1167/iovs.16-19133 (PMC5413215; doi:10.1167/iovs.16-19133)
Supplement: Supplement 1 [file iovs-58-04-25_s01.pdf]

Supplementary figure 1

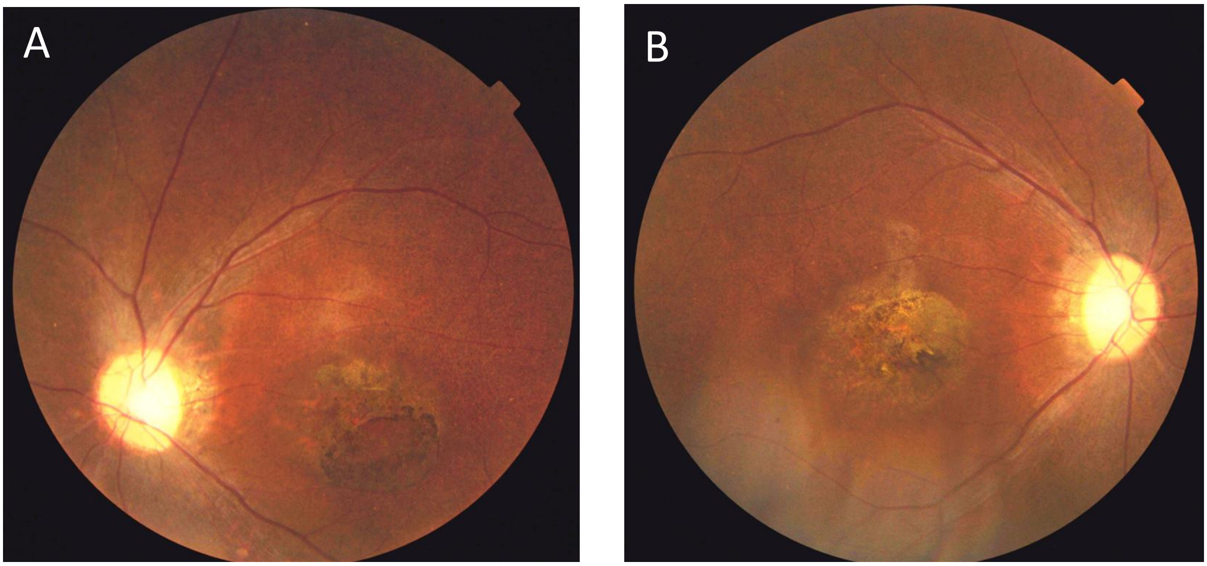

Supplementary figure 1: A and B: Left Macular atrophic lesions with pigment proliferation, characteristic of macular degeneration, can be seen in both eyes.

Supplementary figure 2

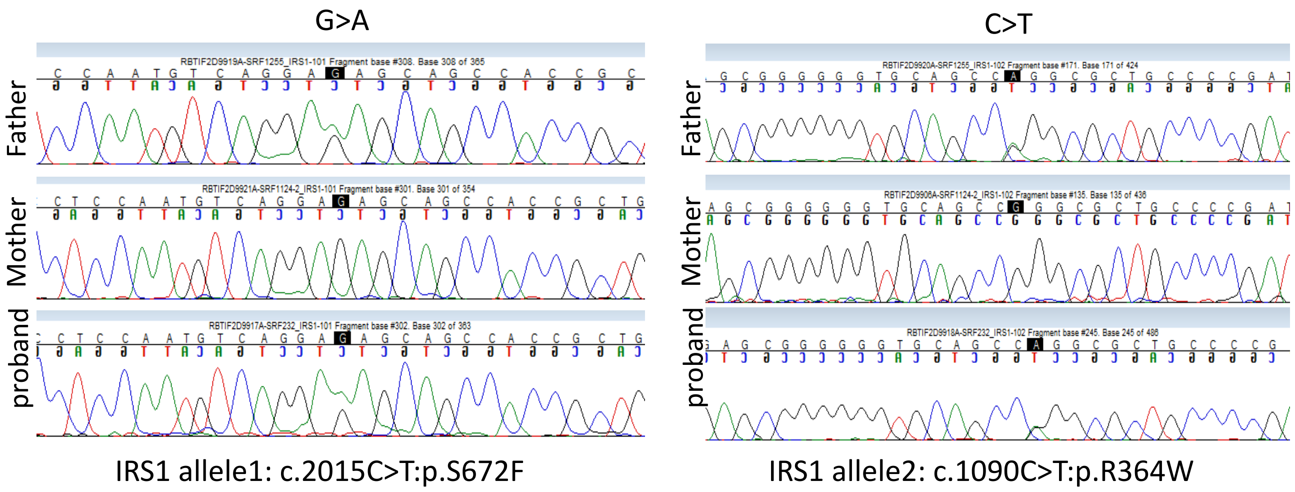

Supplementary figure 2: The alternative candidate alleles in IRS1 gene follow a cis-segregation pattern.

Supplementary figure 3

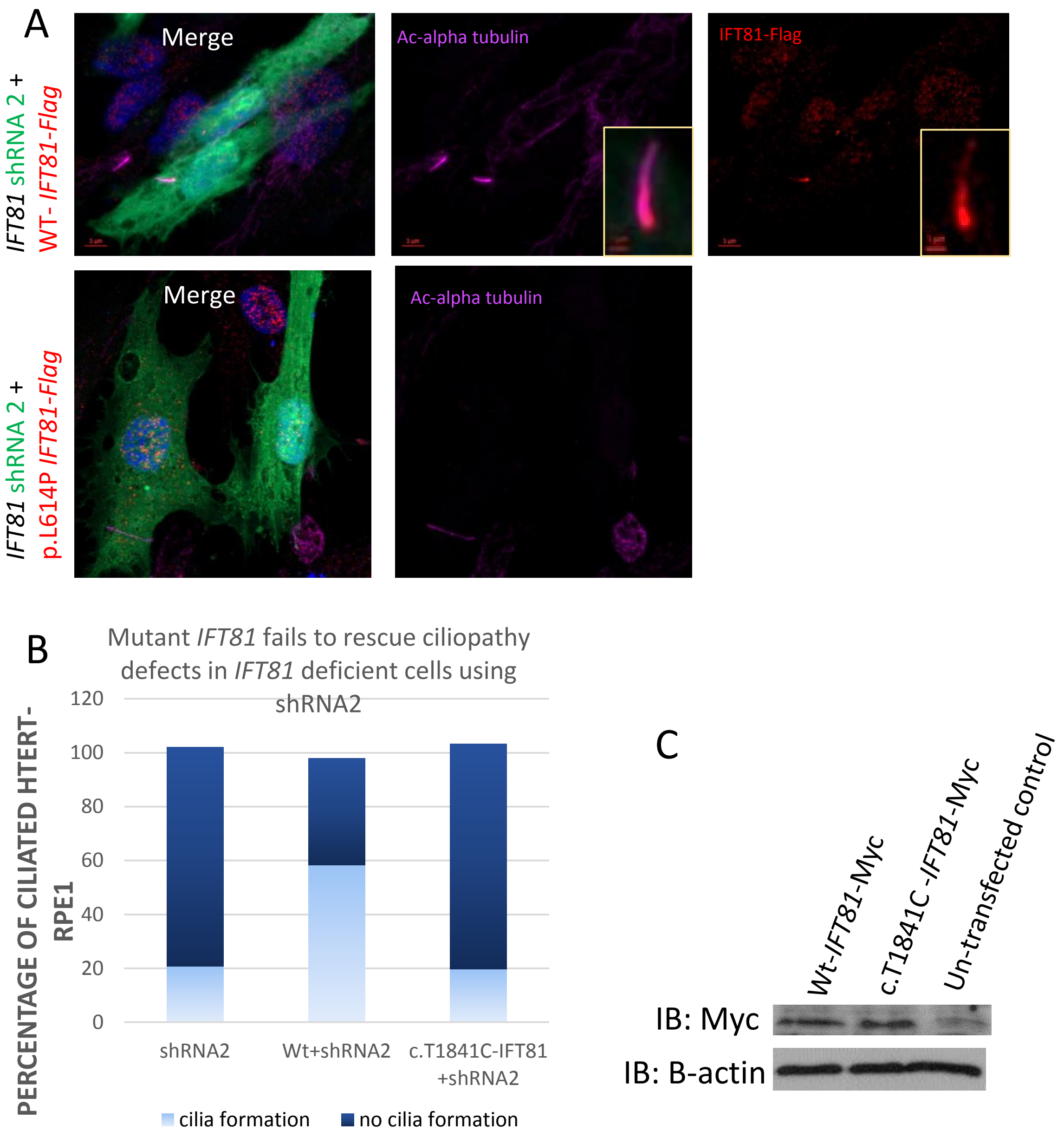

Supplementary figure 3: Lack of rescue potential of *IFT81* mutant in RPE subjected to shRNA 2 based knockdown of endogenous *IFT81.* A. Representative images displaying rescue of ciliogenesis in cells expressing shRNA2 by co-expression of WT IFT81 (top panel). Co-expression of mutant IFT81 does not rescue ciliogenesis (bottom panel). B. Quantification of cells co-expressing shRNA1 and WT or mutant IFT81 and corresponding ciliated cell percentage. C. The expression level of IFT81 wild-type and mutant protein in transfected 293T cell extract.

Supplementary figure 4

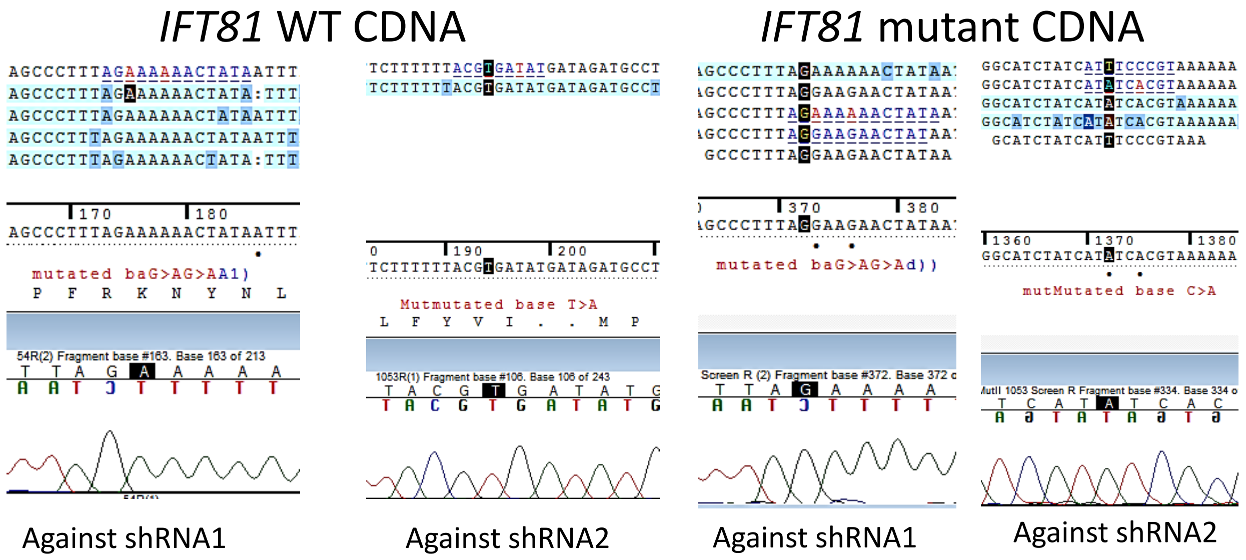

Supplementary figure 4: Synonymous base changes to make shRNA-resistant *IFT81* wild-type and mutant CDNA

Supplementary figure 5

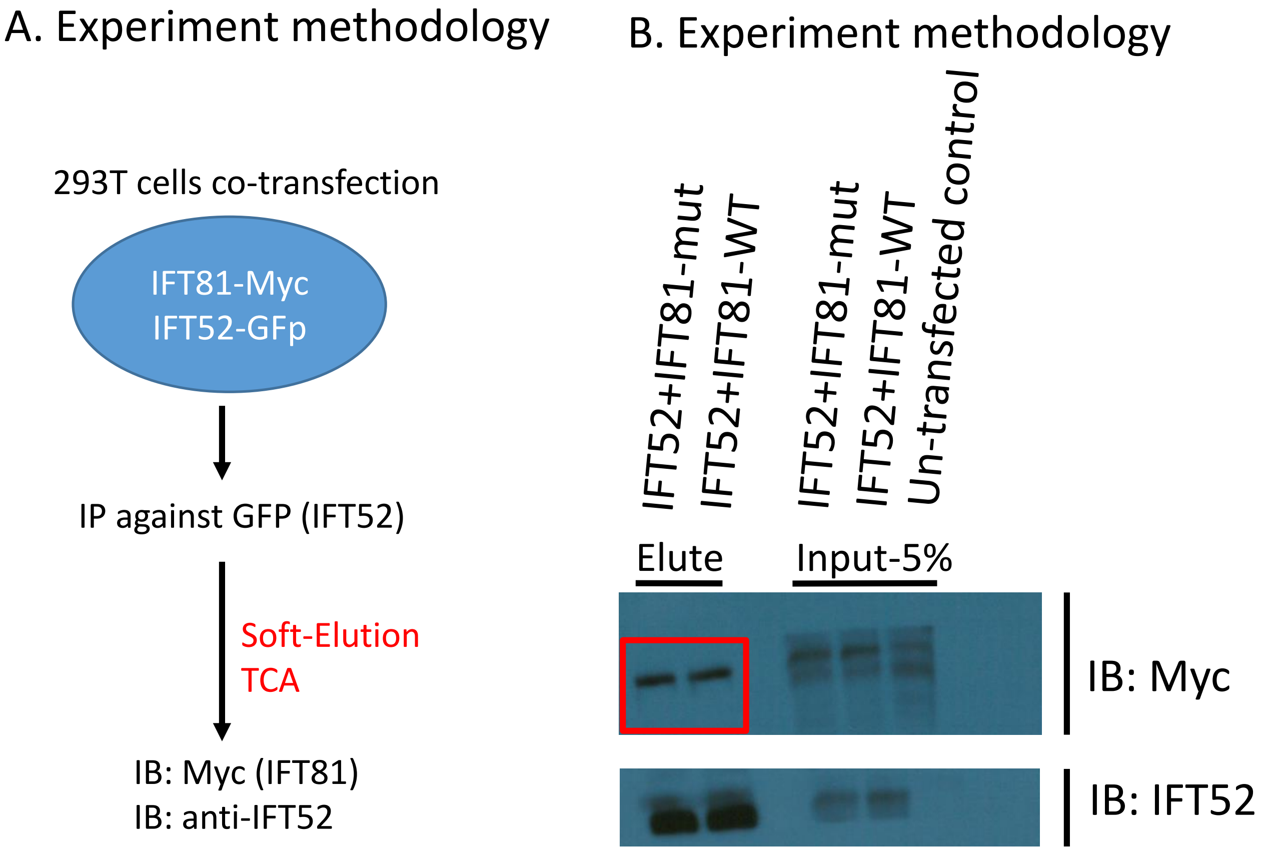

Supplementary figure 5: A. Experimetntal Mthodolgy for Immunopreceipitation and western blotting to confirm interaction with of WT and c.1841T>C mutant allele with IFT52. B. The c.1841T>C mutant allele does not significantly alter the interaction of IFT81 with IFT52/46 sub-complex.
