# Supplementary material for: IFT81 as a Candidate Gene for Nonsyndromic Retinal Degeneration
Source: Invest Ophthalmol Vis Sci. 2017 May;58(5):2483–90. doi: 10.1167/iovs.16-19133 (PMC5413215; doi:10.1167/iovs.16-19133)
Supplement: Supplement 2 [file iovs-58-04-25_s02.pdf]

**Supplementary data**

## *IFT81* as a candidate gene for non-syndromic retinal degeneration.

Rachayata Dharmat[1,2#], Wei Liu[2#], Zhongqi Ge[1,2#], Zixi Sun[3#], Lizhu Yang[3], Yumei Li[1,2], Keqing Wang[1,2], Kandace Thomas[2], Ruifang Sui[3*], Rui Chen[1,2, 4, 5,6*]

**Supplementary methods:**

**Statistical Analysis:**

For both *in* vitro and *in vivo* cell assay, statistical analysis was performed by two-way ANOVA analysis using Graph-pad prism software. In case of *in vivo* assay, statistical analysis was performed by comparing the number of phenotypically null embryo in wild-type and mutant mRNA rescue experiments to the number of phenotypically null zebrafish from control embryos injected with PBS. In case of *in vitro* ciliogenesis assay, the statistical comparison was performed between shRNA-transfected IFT81 knockdown cells to shRNA-transfected cells rescued with wildtype and mutant vector.

**Protein analysis**

For determining IFT protein levels, HEK293T were lysed in RIPA buffer by sonication for 15 seconds at 30% amplitude. Western blotting performed using antibodies against c-myc (clone 9E10, 1:1000; Covance), was developed by chemiluminescence (Pierce ECL western blotting substrate-32106) and imaged using a KODAC X-OMAT 2000 processor (Fujifilm).

**Immunoprecipitation**

For testing interaction between IFT81 mutant allele and IFT52, plasmid constructs carrying flag-tagged *IFT81* wildtype and c.1841T>C mutant allele was co transfected with IFt52-GFP plasmid constructs into HEK-293T cells. Protein extracts were subjected to Immunoprecipitation using GFP-Nanobeads (Chromoteck) and interacting proteins eluted

using TCA soft elusion technique. The elutes were analyzed using anti IFT52 (Gregory Pazour) and Anti-Myc (Covance MMS-150p).

## Supplementary Clinical data

**Supplementary Table 1:** Predicted damaging effect of missense variants observed in the proband.

| Gene | NM_number | Missense variants | SIFT* | Polyphen2_HDIV* | Polyphen2_HVAR* | LRT§ | Mutaiton-taster‡ | Mutation-Assessor† | Allele frequency | Grantham distance# |
|---|---|---|---|---|---|---|---|---|---|---|
| **IFT81** | NM_001143779 | c.1841T>C (p.L614P) | T | D | D | De | Di | M | 0.00001849 | 98 |
| **IRS1** | NM_005544 | c.2015C >T (p.S672F) | T | D | D | De | Di | L | 0 | 155 |
| **IRS1** | NM_005544 | c.1090C>T (p.R364W) | D | D | D | N | Di | L | 0.00001809 | 101 |
| **FAM186A** | NM_001145475 | c.4191G>A (p.M1397I) | T | B | B | . | N | N | 0 | 60 |
| **FAM186A** | NM_001145475 | c.1262G>A (p.R421Q) | T | B | B | . | N | N | 0.00005096 | 54 |
| **PKHD1L1** | NM_177531 | c.3835G>A (p.G1279R) | T | B | B | N | N | M | 0.0000166 | 125 |

| | | | | | | | | | | |
|---|---|---|---|---|---|---|---|---|---|---|
| **PKHD1L1** | NM_177531 | c.8099A>G (p.N2700S) | T | B | B | N | N | N | 0 | 46 |

Footnotes: * - D, damaging, T, tolerant, B, benign; § - De, deleterious, N, Neutral, U, Unknown; ‡ - Di, disease-causing, N, Neutral, P, Polymorphism; † - M, medium damaging (predicted functional), L, low damaging (predicted nonfunctional). '.' in all databases, Not Found; # - Grantham scores (possible range from 5 to 215) of <50 were classified as nonsynonymous conservative, 51 to 100 as moderately conservative, 101 to 150 as moderately radical, and >151 as radical changes[39].

**Supplementary data 2: Patient's test report**

Blood routine, liver function, renal function and blood fat tests were all in normal functional range suggesting lack of any syndromic defects.

**Examination Information & Report:**

**<u>Test: Ultrasound of the Liver, Gallbladder, Pancreas, Spleen, Kidneys</u>**

**Resolutions: 1280*800, 800*1280, 1080*1440**

Date of Examine: 2013/04/18 Date of Report: 2013/04/18

Region of Examine: Liver, Gallbladder, Pancreas, Spleen, Kidneys

Results: The liver edge reaches the bottom of sternum and the bottom of the right libs. The echo from the liver is uniform. The size of gallbladder is normal, and its wall is smooth. We didn't see any gallstone or neoplasia. The diameter of the common bile duct is 0.4cm, the Portal Venous is 1.0cm. The Morphology of the pancreas is normal. The thickness of the spleen is 3.5cm, and we didn't detect it from the bottom of libs. Two kidneys are normal in sizes, morphologies and structure. There's no dilation found in renal pelvis or renal calyx.

**<u>Test: Liver function</u>**

| Tests | Initials | Values | Units | Indicators | Reference Intervals |
|---|---|---|---|---|---|
| **pH** | PH | 7.5 | | | 5.0-8.0 |
| **White Blood Cells** | WBC | 70 | Cell/ul | H | <15 |
| **Nitrite** | NIT | NEG | | | NEG |
| **Protein** | PRO | NEG | g/L | | NEG |

| | | | | | |
|---|---|---|---|---|---|
| **Glucose** | GLU | NEG | mmol/L | | NEG |
| **Ketone** | KET | NEG | mmol/L | | NEG |
| **Urobilinogen** | UBG | 3.2 | umol/L | | 3-16 |
| **Bilirubin** | BIL | NEG | umol/L | | NEG |
| **Red Blood Cell** | BLD | NEG | Cell/ul | | <25 |
| **Density/specific gravity** | SG | 1.025 | | | 1.005-1.030 |

**<u>Test: Kidney function</u>**

| Tests | Initials | Values | Units | Indicators | Reference Intervals |
|---|---|---|---|---|---|
| **Alanine aminotransferase** | ALT | 16 | U/L | | 05 -40 |
| **Total Protein** | TP | 75 | g/L | | 60-85 |
| **Albumin** | Ab | 44 | g/L | | 35-51 |
| **Albumin/Globulin Ratio** | A/G | 1.4 | | | 1.0-2.5 |
| **Total Bilirubin** | Tbil | 8.7 | uMol/L | | 5.1-22.1 |
| **Direct Bilirubin** | Dbil | 2.7 | uMol/L | | 0.0-8.6 |
| **Glutamyl Endopeptidase** | GGT | 33 | U/L | | 10 -67 |
| **Alkaline phosphatase** | ALP | 56 | U/L | | 30-120 |
| **Aspartate Aminotransferase** | AST | 15 | U/L | | 05 -37 |
| **Total Bile Acid** | TBA | 3 | uMol/L | | 0.1-10.0 |
| **Lactate Dehydrogenase** | LD | 114 | U/L | | 97-270 |
| **Cholinesterase** | ChE | 9.1 | kU/L | | 4.0-12.6 |
| **Potassium** | K | 4.4 | mmol/L | | 3.5-5.5 |

| | | | | | |
|---|---|---|---|---|---|
| **Sodium** | Na | 143 | mmol/L | | 134-145 |
| **Chlorine** | Cl | 108 | mmol/L | | 96-111 |
| **Total carbo dioxide** | TCO2 | 27.1 | mmol/L | | 20.0-34.0 |
| **Calcium** | Ca | 2.4 | mmol/L | | 2.13-2.70 |
| **Creatinine** | Cr(E) | 65 | mmol/L | | 45-84 |
| **Urea** | Urea | 4.06 | mmol/L | | 1.07-7.14 |
| **Glucose** | Glu | 4.9 | mmol/L | | 3.6-6.1 |
| **Uric Acid** | UA | 371 | mmol/L | H | 150-357 |
| **Phosphate** | P | 1.33 | mmol/L | | 0.81-1.45 |
| **Adenosine dehydrogenase** | ADA | 11.6 | U/L | | 4.0-24.0 |
| **Pre Albumin** | PA | 238 | mg/L | | 180-400. |

**Test: Blood Routine**

| Tests | Initials | Values | Units | Indicators | Reference Intervals |
|---|---|---|---|---|---|
| **White Blood Cell** | WBC | 7.82 | *1059/L | | 4.0-10.0 |
| **Lymphocyte Percentage** | LY% | 35.3 | % | | 20-40.0 |
| **Monocyte Percentage** | MONO% | 4.2 | % | | 3.0-8.0 |
| **Neutrophil Percen** | NEUT% | 57.2 | % | | 50.0-75.0 |

| | | | | | |
|---|---|---|---|---|---|
| **Eosinophil Percen** | EOS% | 3.2 | % | | 0.5-5.0 |
| **Basophil Percentage** | BASO% | 0.1 | % | | 0.0-1.0 |
| **Lymphocyte Count** | LY# | 2.76 | *1059/L | | 0.8-4.0 |
| **Monocyte Count** | MONO# | 0.33 | *1059/L | | 0.12-0.80 |
| **Neutrophil Count** | NEUT# | 4.47 | *1059/L | | 2.0-7.5 |
| **Eosinophil Count** | EOS# | 0.25 | *1059/L | | 0.02-0.50 |
| **Basophil Count** | BASO# | 0.01 | *1059/L | | 0.0-0.1 |
| **Red Blood Cell** | RBC | 4.38 | *10512/L | | 3.5-5.0 |
| **Hemoglobin** | HGB | 132 | g/L | | 110-150 |
| **Hematocrit** | HCT | 38.9 | % | | 35.0-50.0 |
| **Mean Corpuscular Volume** | MCV | 88.8 | fl | | 82.0-97.0 |
| **Mean Corpuscular Hemoglobin Concentration** | MCHC | 339 | g/L | | 320-360 |
| **Mean Corpuscular Hemoglobin** | MCHC | 30.1 | pg | | 27.0-32.0 |
| **Red Blood Cell Distribution Width** | RDW-S | 39.7 | fl | | 39.0-46.0 |
| **Red Blood Cell Distribution Volume** | RDW-C | 12.4 | % | | 0.0-15.0 |
| **Platelet** | PLT | 225 | *1059/L | | 100-300 |
| **Platelet Hematocrit** | PCT | 0.26 | % | | 0.11-0.28 |
| **Platelet Distribution Width** | PDW | 14.3 | % | | 9.0-17.0 |
| **Mean Platelet Volume** | MPV | 11.7 | fl | | 7.0-13.0 |
| **Large Platelet Ratio** | P-LCR | 39.3 | % | | 13-43 |
